# Impact of motor task conditions on end-point kinematics and trunk movements during goal-directed arm reach

**DOI:** 10.1038/s41598-024-54723-4

**Published:** 2024-02-24

**Authors:** Bokkyu Kim, Jaimie Girnis, Vanessa Sweet, Tobias Nobiling, Tarek Agag, Christopher Neville

**Affiliations:** 1 Department of Physical Therapy Education, College of Health Professions, SUNY Upstate Medical University, Syracuse, NY 13066 USA; 2 Jones Memorial Hospital, Wellsville, NY USA; 3 University of Rochester Medical Center, Rochester, NY USA

**Keywords:** Motor control, Rehabilitation

## Abstract

Task conditions significantly impact human motor control. We investigated how task type, difficulty, and constraints influence the kinematics of goal-directed arm reaching. Non-disabled young adults performed two distinct goal-directed arm reaching tasks: pointing and picking up an object with chopsticks. These tasks were carried out under various conditions, including constrained and unconstrained elbow extension and two different task difficulties. We collected kinematic data using a 3-D motion capture system and analyzed the effects of different task conditions on kinematic variables using linear mixed-effects regression analysis. Our findings revealed statistically significant differences in kinematics between the two tasks. Arm reaching during the picking-up task was slower and exhibited jerkier movements compared to the pointing task. Additionally, when arm reaching was performed with constrained elbow extension, it led to slower and jerkier movements, with an increased involvement of trunk movements compared to the unconstrained condition. These findings show that complex manipulative motor tasks requiring higher hand dexterity necessitate feedback-based control of arm reaching, but simple pointing tasks requiring less hand dexterity do not. In conclusion, our study sheds light on the influence of task conditions on goal-directed arm reaching kinematics and provides valuable insights into the motor control strategies involved in different tasks.

## Introduction

Individual, task, and environmental factors interact to define the majority of motor control strategies^1^. Research on goal-directed arm reaching movement strategies has extensively explored their correlation with various task conditions. For instance, it has been observed that the speed of arm reaching movements differs when grasping smaller objects compared to larger ones^2^, a phenomenon commonly attributed to the accuracy-speed trade-off, a well-established principle known as Fitts’ law, which delineates the impact of task difficulty on performance speed^3,4^. Additionally, studies have highlighted the influence of task difficulty on multi-joint coordination^5^. Furthermore, the position of the target emerges as a significant factor affecting arm reaching kinematics in activities involving arm reaches^6^. Gordon and colleagues^6^ identified that target locations in goal-directed arm reaching tasks exhibit distinct arm reaching kinematic features, such as initial acceleration and direction-dependent end-point variability, influenced by different limb inertial resistance attributable to target direction.

Likewise, previous research has shown that different motor task types can alter the speed of goal-directed arm reaching^7,8^. When people perform a reaching and grasping task, arm reaching is slower than when they perform a reaching and pointing task. Slower arm reaching movements in the object grasping task than in the pointing task can be explained by different motor control strategies for different types of tasks. Prior research indicates that individuals performed the picking up task with greater reliance on feedback-based control compared to the pointing task^7,9^. Carnahan and colleagues demonstrated that there is no difference in time to peak velocity of the arm reaching movement between pointing and grasping tasks, while there was a greater amount of time to decelerating arm reaching when grasping an object as opposed to pointing (i.e., longer deceleration tail of the tangential velocity curve)^7^. This result may suggest that individuals might employ greater arm deceleration as they approach the target to improve the precision of the hand position required to execute the grasping action.

Previous studies have predominantly focused on fundamental reaching and grasping tasks, overlooking the importance of motor skills required for activities demanding higher levels of hand dexterity, such as small object manipulation or tool use. It has been well studied that the use of novel tool can affect the hand kinematics despite the extensive literature on reaching and grasping, specifically hand kinematics^10^, there is a noticeable gap in our understanding of the difference in arm reaching kinematics associated with tasks requiring different levels of hand dexterity^7^. This gap hinders our comprehension of motor control strategies employed by individuals in daily life activities, especially in people with arm and hand motor deficits due to neurologic disorders, such as stroke. Moreover, although prior research has mostly examined the influence of motor task conditions on the kinematics of arm reaching, only a limited number of studies have explored how these task conditions affect the trunk movement that occurs during goal-directed arm reaching. After stroke, compensatory trunk movements are commonly established to substitute for the reduced range of motion in the shoulders and elbows, when stroke survivors perform goal-directed arm reaches^11–13^. Gaining insight into how task variables impact trunk movement during arm reaching is crucial to developing rehabilitation strategies and assessments that specifically target reducing the compensatory trunk movement during arm reaches in stroke survivors.

The motivation behind our study stems from the need to bridge this gap in the scientific literature by comparing arm reaching kinematics in different tasks involving different levels of hand dexterity demand. By exploring these tasks, we investigated the distinct motor control strategies underlying goal-directed arm reaching in situations that reflect activities that are used in daily life, thus contributing novel insights into motor control and skill acquisition. Through this research, we seek to enhance our understanding of motor behavior in non-disabled young adults, with potential implications for stroke rehabilitation.

The primary aim of this study was to determine how task difficulty and type impacted the kinematics of goal-directed arm reaching in non-disabled young adults using chopstick operation tasks. We predicted that when task difficulty and hand dexterity requirements increased, feedback-based online adjustment of goal-directed arm reaching would also increase. Secondarily, this study also investigated the impact of task conditions on compensatory trunk movements during goal-directed arm reaches. We speculated that increased task difficulty and hand dexterity requirement would also increase trunk compensation in non-disabled young adults. Finally, we investigated how non-disabled young adults employ compensatory trunk movements during goal-directed arm reaches when the elbow extension of the performing arm is constrained. We hypothesized that, under the elbow extension constraint, non-disabled young adults would adopt a motor control strategy characterized by slower arm reaching and an increased reliance on trunk movements compared to the no-constraint condition. This hypothesis stems from the expectation that the imposition of an elbow movement constraint would prompt an immediate adjustment in their motor control strategy.

## Methods

### Study design and participants

This study was a cross-sectional basic human motor control study with a one-time visit to a motion analysis research lab at SUNY Upstate Medical University. We recruited 11 non-disabled young adults between 24 and 27 years old with no history of neurologic or musculoskeletal conditions affecting their ability to perform daily activities. Participants were screened using a questionnaire to determine their eligibility. The demographics of participants are summarized in Table 1. The SUNY Upstate Medical University Institutional Review Board (IRB) approved the study protocol. All experimental procedures were performed in accordance with the relevant guidelines and regulations.

**Table 1:** Non-disabled young participants' demographics and chopstick usage history and self-efficacy.

Demographic variables	Statistics
Age (mean ± SD [Min − Max])	25.09 ± 1.22 [24–27]
Sex (F/M)	7/4
Race (white)	11
Hand dominance (right/left)	9/2
Previous chopstick usage (yes/no)	8/3
Chopstick usage frequency within 6 months	
1–2 times per month (counts)	3
Less than 5 times (counts)	5
Chopstick skill self-efficacy (mean ± SD [Min − Max]), with a scale ranging from 0 (not confident at all to pick up a given object) to 100 (absolutely confident to pick up a given object)	
Dominant hand—large object	46.91 ± 39.52 [0–100]
Non-dominant hand—large object	19.73 ± 24.65 [0–64]
Dominant hand—small object	43.73 ± 34.56 [0–79]
Non-dominant hand—small object	14.82 ± 17.06 [0–48]

### Experimental procedure

During the lab visit, the participants were asked to complete the informed consent and screened to determine eligibility. For non-disabled adults, we used the Quick Disabilities of Arm, Shoulder, and Hand Outcome (QuickDASH) Questionnaire to screen their eligibility. Eligible participants were asked to complete several questionnaires regarding their demographics, hand dominance, previous chopstick use experience, and self-efficacy using chopsticks. After completing the questionnaires, participants performed goal-directed arm reaching tasks with different conditions. At the end of the experiment, participants completed an intrinsic motivation inventory for motor task performance.

#### Questionnaires for chopstick motor skill

We performed psychosocial cognitive-behavioral assessments, including chopstick experience, chopstick skill self-efficacy, and intrinsic motivation for chopstick skill performance, using questionnaires in REDCap, an online survey platform. Chopstick skill self-efficacy was assessed for each hand through a self-reported numerical scale ranging from 0 to 100. Participants were asked to rate their confidence in using chopsticks to pick up a given object with both their left and right hands on this scale. The numerical values ranged from 0, indicating 'Not confident at all,' to 100, representing 'Absolutely confident.' This assessment aimed to comprehensively capture participants' perceived ability and confidence in chopstick manipulation, allowing for a detailed examination of self-efficacy levels for each hand separately. The numerical scale provided a quantitative means to assess and compare individual levels of self-efficacy, facilitating a nuanced understanding of participants' perceived proficiency in chopstick use for each hand. The impact of these variables on the relationship between task conditions and arm reaching kinematics was determined by analyzing the outcomes of psychosocial cognitive-behavioral factors.

#### Arm and hand motor tasks

**Figure 1:**
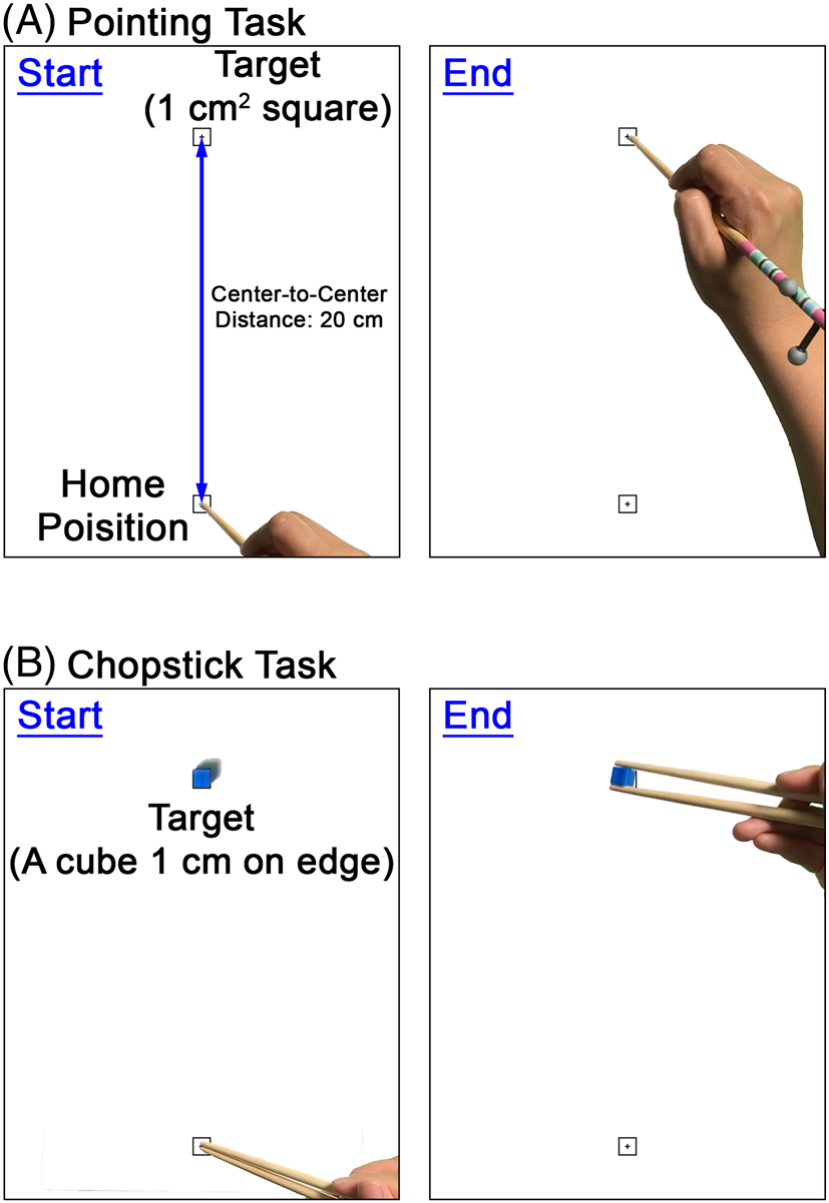
Pointing task and chopstick object pick up task with a large target. (**A**) Pointing task. Participants hold a chopstick and place the tip on the home position. When the cue is given, participants reach and tap the target with the chopstick tip as quickly as possible. (**B**) Chopstick task. Participants hold a pair of chopsticks and locate the tips on the home position. When the cue is given, participants reach and grasp the target object with a pair of chopsticks.

We utilized two upper extremity motor tasks: (1) arm reaching and *pointing to a target* with a chopstick and; (2) arm reaching and *picking up an object* with a pair of chopsticks. We had two different target sizes in each task type (large target and small target). Details of the motor task conditions, including task difficulty and type, are described in our previous protocol paper^14^ (Fig. 1).

Participants performed these tasks in two different electrical stimulation conditions: (1) motor level stimulation for dominant elbow flexors; (2) sensory level stimulation for the non-dominant forearm.

The purpose of the motor-level electrical stimulation to the elbow flexors of the dominant arm is to constrain elbow extension and to test whether the elbow extension constraint is associated with the use of compensatory movement strategies in non-disabled adults. In the sensory level stimulation condition, participants performed the tasks with sensory level stimulation to the dorsal surface of the non-performing forearm. The sensory stimulation did not affect the participants' motor performance, but served as a sham condition for motor-level stimulation.

Participants performed those tasks in a sitting position in front of a table. The target location was placed at 80% of the participant's maximum arm reaching distance without trunk movement during the preparation. Participants were instructed to maintain an upright trunk posture at the beginning of each performance. Trunk movement was not constrained during task performance. Before performing the task, an investigator instructed the participant using a demonstration video to standardize chopstick use.

Participants were instructed to hold a chopstick with their dominant hand (non-disabled adults) for pointing tasks. A laminated task template paper was placed and fixed on the table. The template paper had two squares with a center cross (home position and target location). The target location was 20 cm in front of the home position. The task goal was to reach and tap the center of the target location with the chopstick tip as quickly and accurately as possible.

Participants were instructed to hold a pair of chopsticks with their dominant hand for object pick-up tasks using chopsticks. The same task template paper was placed and fixed on the table. The target object was placed at the target location. The target objects were a wooden block 10 mm on the edge for the easier task and a plastic block 3 mm on the edge for the more difficult task. The task goal was to reach and pick up the target object to a 1-inch height using a pair of chopsticks as quickly as possible.

The cue for the task was electrical stimulation either on the non-performing forearm or the performing upper arm, depending on the task condition. The electrical stimulation was applied for three seconds. Participants were informed to complete the task while the electrical stimulation was on. They performed three familiarization trials and ten actual trials for each condition. There was a 3-s break between each trial. If the participant could not complete the task within three seconds, they were asked to bring the chopstick(s) back to the home position and ready for the next trial. There was a 2-min break between each task condition. Task conditions are summarized in Table 2.

**Table 2:** Summary of task conditions.

No elbow extension constraint			Elbow extension constraint		
	Task type (hand dexterity requirements)			Task type (hand dexterity requirements)	
Target size (difficulty)	Tapping large target (less dexterity and easy)	Picking up large object (more dexterity and easy)	Target size (difficulty)	Tapping large target (less dexterity and easy)	Picking up large object (more dexterity and easy)
	Tapping small target (less dexterity and difficult)	Picking up small object (more dexterity and difficult)		Tapping small target (less dexterity and difficult)	Picking up small object (more dexterity and difficult)

#### Electrical stimulation for elbow extension restriction and movement cueing

A neuromuscular electrical stimulation (NMES) unit was used for this study. Electrical stimulation at the motor level of the elbow flexors was utilized to restrict elbow extension during motor performance. The forearm electrical stimulation at the sensory level was utilized as a cue to initiate the movement. A pair of 3" round carbon-impregnated electrodes with wet sponges were placed on the elbow flexor's motor points for motor-level stimulation. A pair of disposable adhesive electrodes were placed on the non-performing forearm for sensory-level stimulation. A symmetric biphasic pulsed waveform was utilized. The frequency of the electrical stimulation was 35 pulses per second (PPS) to induce complete tetanic muscle contraction but to minimize muscle fatigue. Pulse durations were set at 300 milliseconds (ms) for motor-level stimulation and 50 ms for sensory-level stimulation, respectively. This pulse duration setting was based on the strength-duration curve that shows the relationship between pulse duration and intensity for sensory and motor thresholds. A shorter pulse duration for sensory level stimulation provided more precise control of the intensity for sensory stimulation without muscle contraction. Stimulation intensities were determined before participants performed the motor tasks. Sensory level stimulation was set to an intensity where participants could feel 10 out of 10 stimulations without muscle contraction. Motor level stimulation was set to an intensity that generated 90 degrees of elbow flexion from a seated position with full elbow extension. All the participants were able to tolerate the motor-level electrical stimulations. One second of ramp up and down was set for participant comfort.

#### Kinematic data recording

The optical camera motion capture system recorded the goal-directed arm reaching kinematics during motor performance. We utilized a 10-camera Vicon optical 3D motion capture system (Vantage Cameras, Vicon, Oxford, UK) with The Motion Monitor software (Version of XGen 3.1, The Motion Monitor, Inc., Chicago, IL, USA). Passive reflective motion capture markers were attached to the participant's upper extremities (lateral side of the upper arm, lateral side of the forearm, over the third metacarpal bone) and posterior aspect of the upper trunk (between scapulae). Using the Motion Monitor software, we digitized the participant's body segments using an upper extremity trunk biomechanical model with X segments. This process allows us to track specific upper extremity and trunk segments, such as hand, wrist joints, elbow joints, shoulder joints (i.e., glenohumeral joints), and seventh cervical bone. A marker set was also attached to a chopstick to digitize the position of the chopstick's tip. Another marker set was placed on the table to digitize the object's home and target positions. Kinematic data were recorded at 100 frames per second (fps). More details on the motion capture system preparation and kinematic data collection are described in our protocol paper^14^.

### Kinematic data analysis

Motion capture data were exported from the data acquisition software to a text file for each task condition for each participant. The exported data included position data for the following locations and body segments: (1) the tip of a chopstick in the x, y, and z axes; (2) the home and target locations of the template paper on the table; (3) hands (center of third metacarpal bone); (4) elbow joints; (5) glenohumeral joints; (6) trunk (C7 vertebra).

The position data were processed using a custom MATLAB script. First, the raw position data were filtered using a 3rd-order Butterworth low-pass filter with a 3 Hz cutoff. For endpoint kinematics, we utilized the filtered position data of the hand. The tangential velocity of the hand was calculated as the first derivative of the resultant of the 3-dimensional hand positions.

Using each trial's tangential velocity profile, movement onset and offset were determined. Movement onset was defined as the first frame of the reaching, where the tangential velocity is above 0.01 m/s. Movement offset was defined as the last frame of the reaching, where tangential velocity is above 0.01 m/s. The peak tangential velocity was also determined as the maximum tangential velocity amplitude of the velocity profile that exceeds the amplitude of 0.2 m/s, and the time interval between 2 peaks must be at least 3 s^15^, given that there was a 3-s break between trials.

Based on the movement onset, offset, and peak tangential velocity, we calculated the following kinematic variables of goal-directed arm reaches: movement duration (MD), peak tangential velocity amplitude (PV), absolute time to peak velocity (aTTPV), relative time to peak velocity (rTTPV), peak tangential acceleration amplitude (PA), absolute time to peak acceleration (TTPA), log dimensionless jerk (LDJ), trunk displacement (TD), and shoulder trajectory length (STL).

MD was calculated as the time difference between movement onset and offset. Peak velocity amplitude was the maximum velocity during the reaching movement. Absolute time to peak velocity (aTTPV) was the time difference between movement onset and peak velocity^15^. Relative time to peak velocity (rTTPV) was the percentage of aTTPV relative to movement duration^15^. Absolute time to peak acceleration (TTPA) was the time difference between movement onset and peak acceleration^15^. Log dimensionless jerk (LDJ) was calculated using the third derivative from the resultant of the 3D hand positions^16^. We calculated the log dimensionless jerk to represent the smoothness of the goal-directed arm reaching trajectory^16^. Trunk compensatory movements were measured using two different measures: (1) trunk displacement (TD)^11,12^ and (2) shoulder trajectory length (STL)^14^. We calculated TD between movement onset and offset using the trunk (C7) landmark position. STL was calculated as the shoulder landmark travel distance between the reaching movement onset and offset. Our preliminary data analysis showed that this shoulder landmark travel distance is more sensitive to trunk movement during arm reaching as it captures the trunk movement in the anterior–posterior, mediolateral, and superior-inferior directions.

Since determining the effect of task conditions on goal-directed arm reaching strategies was the primary objective of this research, we limited our analysis to the performing hand's goal-directed arm reaching movement. The performing hand was utilized as the endpoint for endpoint kinematics. An analysis of the finger movement kinematics during item manipulation with a set of chopsticks was not conducted.

### Statistical analysis

We performed linear mixed-effects regression^17^ to model goal-directed arm reaching kinematics and compensatory trunk movements as a function of task type (pointing vs. picking up), task difficulty (small vs. large targets), and elbow extension constraint condition (no constraint vs. constraint). We used each individual trial as a single data point, rather than using an average of 10 trials for each condition for each participant due to the small sample size of the study.

For each kinematic variable, the model included task type, task difficulty, elbow constraint condition, and interactions among these three variables (type*difficulty, type*constraint, difficulty*constraint) as fixed effects terms. All the fixed effects variables were set as categorical variables. Dependent kinematic variables were set as continuous variables. We also included random intercepts and random slopes for individual participants and individual trials to consider the model's inter-individual and inter-trial variability.

We examined residuals for normality and for identifying outliers. We performed statistical analyses using MATLAB and Statistics and Machine Learning Toolbox Release R2020b (MathWorks, Inc., Natick, Massachusetts, United States) with the 'fitlme' function. Models with different random slope and intercept terms were compared to minimize fitting error and the number of model parameters using the 'compare' function. Statistical significance was set at P < .05.

## Results

A likelihood-ratio test indicated that the model, including individual participants and individual trials as random intercepts and slopes, provided a better fit for the data than a model without these random variables. Details of model comparison statistics are summarized in the Supplementary document S1.

### Movement duration (MD)

Examination of the summary output for the entire model indicated that 'task type,' 'elbow extension constraint,' 'task type*task difficulty,' and' task type*elbow constraint' were statistically significant factors of movement duration. Task difficulty alone was not statistically significant.

**Figure 2:**
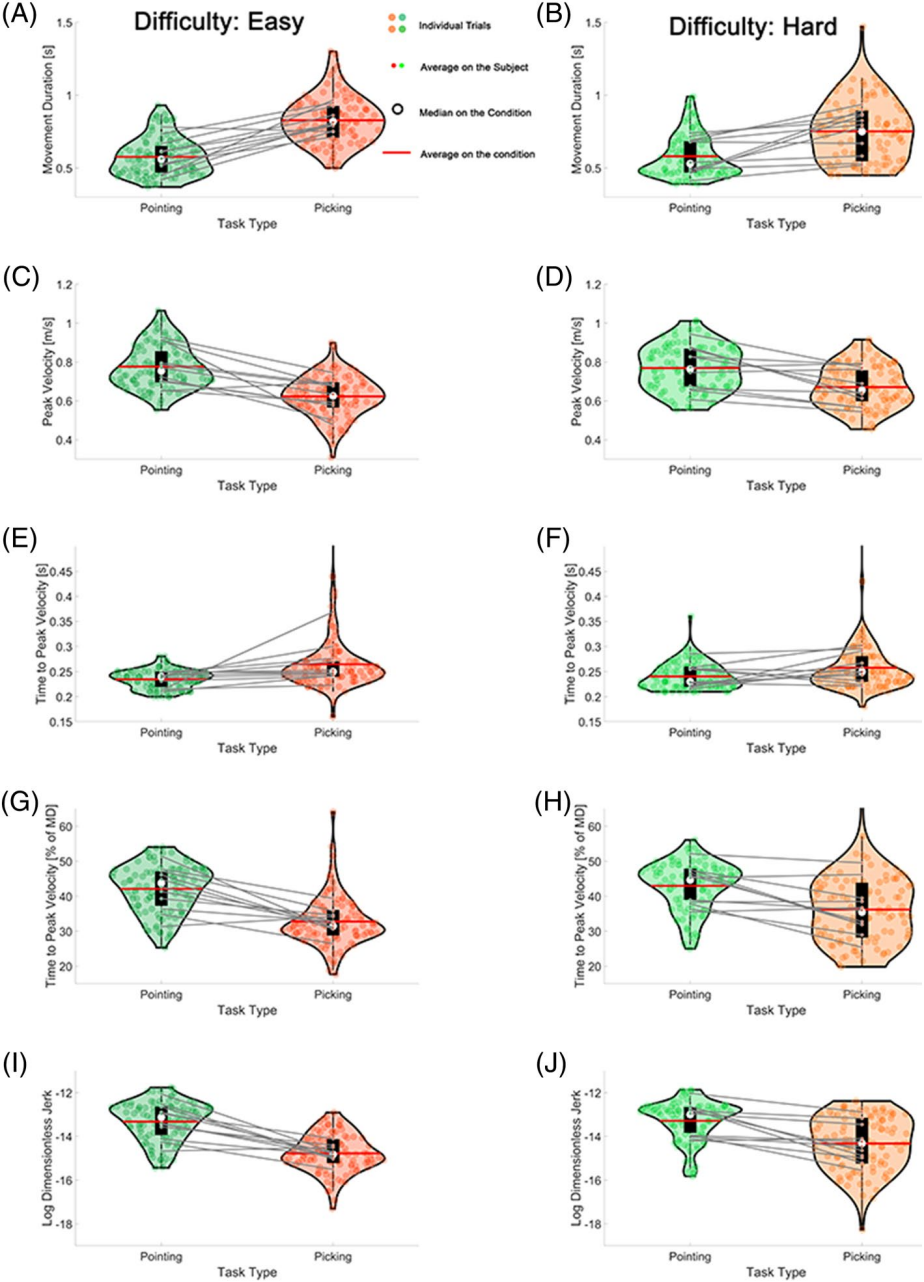
Difference in endpoint kinematics between pointing and picking up tasks. (**A**,**C**,**E**,**G**,**I**) Comparisons of kinematic variables between two tasks with larger target (easy task difficulty). (**B**,**D**,**F**,**H**,**J**) Comparison of kinematic variables between two tasks with smaller target (hard task difficulty).

**Figure 3:**
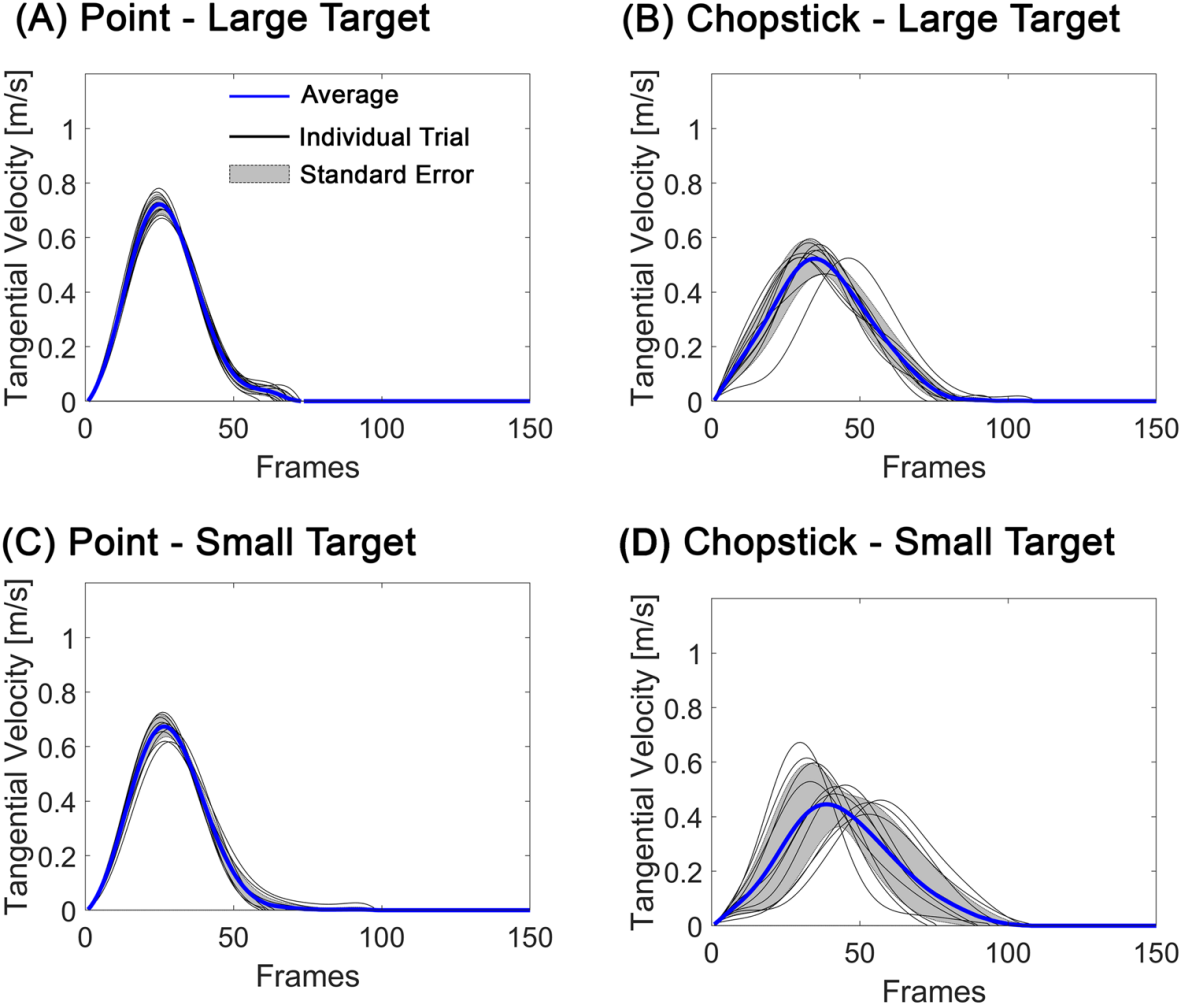
Comparison of velocity profiles between different task conditions. (**A**) Pointing task for a large target. Individual trials are represented by thin black lines, the average of all trials by a thick blue line, and the standard deviation of all trials by a dark shade. (**B**) Chopstick object pick-up task for a large target. (**C**) Pointing task for a small target. (**D**) Chopstick object pick-up task for a small target. The sampling rate of the motion capture system was 100 frames per second.

MD was, on average, an estimated 0.27 s longer in the picking up tasks than in pointing tasks ($\widehat{\beta }=0.27, SE=0.03, t=7.97, p<.001$). Further, MD was, on average, an estimated 0.06 s longer in the elbow extension constraint condition than non-constraint condition ($\widehat{\beta }=0.06, SE=0.03, t=2.43, p=.02$) (Figs. 2A,B, and 3A–D) The interaction term 'task type * task difficulty' displayed a negative coefficient of − 0.11, suggesting that the joint impact of task type and target size is linked to a reduction in movement duration. For example, the duration of arm reaching movement for the picking up task targeting a smaller object was longer than that for the pointing task directed at a larger target. Similarly, the interaction term 'task type * elbow constraint' also showed a negative coefficient of -0.06, indicating that the combined effect of task type and the elbow constraint condition is associated with a decrease in movement duration.

### Peak velocity (PV)

Like MD, task type, elbow extension constraint, the interaction between task type and difficulty, and interaction between task type and elbow extension constraint were statistically significant factors of the PV. PV was, on average, an estimated 0.16 m/s slower in the picking up tasks than in pointing tasks ($\widehat{\beta }=-0.16, SE=0.03, t=-5.88, p<.001$). Besides, PV was, on average, an estimated 0.06 m/s slower in the elbow constraint condition than non-constraint condition ($\widehat{\beta }=-0.06, SE=0.02, t=-3.60, p<.001$) (Figs. 2C,D, and 3A–D) The interaction term 'task type * task difficulty' exhibited a positive coefficient of 0.06, signifying that the collective impact of task type and target size is linked to an elevation in peak velocity. Additionally, the interaction term 'task type * elbow constraint' displayed a positive coefficient of 0.03, indicating that the joint effect of task type and the elbow constraint condition is associated with an increase in peak velocity.

### Absolute time to peak velocity (aTTPV) and relative time to peak velocity (rTTPV)

Like MD and PV, aTTPV was also explained by the same fixed effects terms and interactions. aTTPV was, on average, an estimated 0.04 s longer in the picking up tasks than pointing tasks ($\widehat{\beta }=0.04, SE=0.01, t=3.07, p<.01$). aTTPV was also, on average, an estimated 0.04 s longer in the elbow constraint condition than in the non-constraint condition ($\widehat{\beta }=0.04, SE=0.006, t=3.53, p<.001$) (Fig. 2E,F) The interaction term 'task type * task difficulty' demonstrated a negative coefficient of − 0.02, suggesting that the joint influence of task type and target size is linked to a decrease in absolute time to peak velocity. Similarly, the interaction term 'task type * elbow constraint' also exhibited a negative coefficient of − 0.02, signifying that the collective impact of task type and the elbow constraint condition corresponds to a decrease in absolute time to peak velocity.

For rTTPV, only task type and interaction between the task type and task difficulty were statistically significant. Peak velocity was reached earlier in the picking up tasks than in the pointing tasks on average, with an estimated 9.48% of movement duration ($\widehat{\beta }=-9.48, SE=1.21, t=-7.84, p<.001$) (Fig. 2G,H) The interaction term 'task type * task difficulty' revealed a positive coefficient of 2.90, indicating that the combined impact of task type and target size is associated with an increase in relative time to peak velocity.

### Peak acceleration (PA) and time to peak acceleration (TTPA)

Peak tangential acceleration (PA) and absolute time to peak acceleration (TTPA).

Like MD, PV, and aTTPV, task type, elbow constraint condition, the interaction between target type and size, and interaction between task type and elbow constraint condition were significant main factors of PA and TTPA.

PA was, on average, an estimated 1.28 m/s^2^ less in the picking up task than in the pointing task (${\widehat{\beta }=-1.28, SE=0.09, t=-14.69, p<.001}$). PA was also, on average, an estimated 0.65 m/s^2^ less in the elbow restricted condition than in the non-restricted condition ($\widehat{\beta }=-0.65, SE=0.16, t=-4.06, p<.001$).

TTPA was, on average, an estimated 0.019 s longer in the picking up task than in the pointing task ($\widehat{\beta }=0.019, SE=0.005, t=3.84, p<.001$). TTPA was also, on average, an estimated 0.024 s longer in the condition with elbow constraint than in the condition without the elbow constraint ($\widehat{\beta }=0.024, SE=0.005, t=4.96, p<.001$).

### Log-dimensionless Jerk (LDJ)

The complete model indicated that only task type, the interaction between the task type and difficulty, and the interaction between the task type and elbow constraint condition were significant factors of LDJ. On average, LDJ was an estimated 1.55 lower in the picking up tasks than in the pointing tasks ($\widehat{\beta }=-1.55, SE=0.12, t=-13.43, p<.001$) (Fig. 2I,J) The interaction term 'task type * task difficulty' displayed a positive coefficient of 0.62, indicating that the combined impact of task type and target size is associated with an increase in jerkiness, suggesting less smooth movement. Similarly, the interaction term 'task type * elbow constraint' showed a positive coefficient of 0.28, signifying that the joint effect of task type and the elbow constraint condition is linked to an increase in jerkiness.

### Compensatory trunk movements

**Figure 4:**
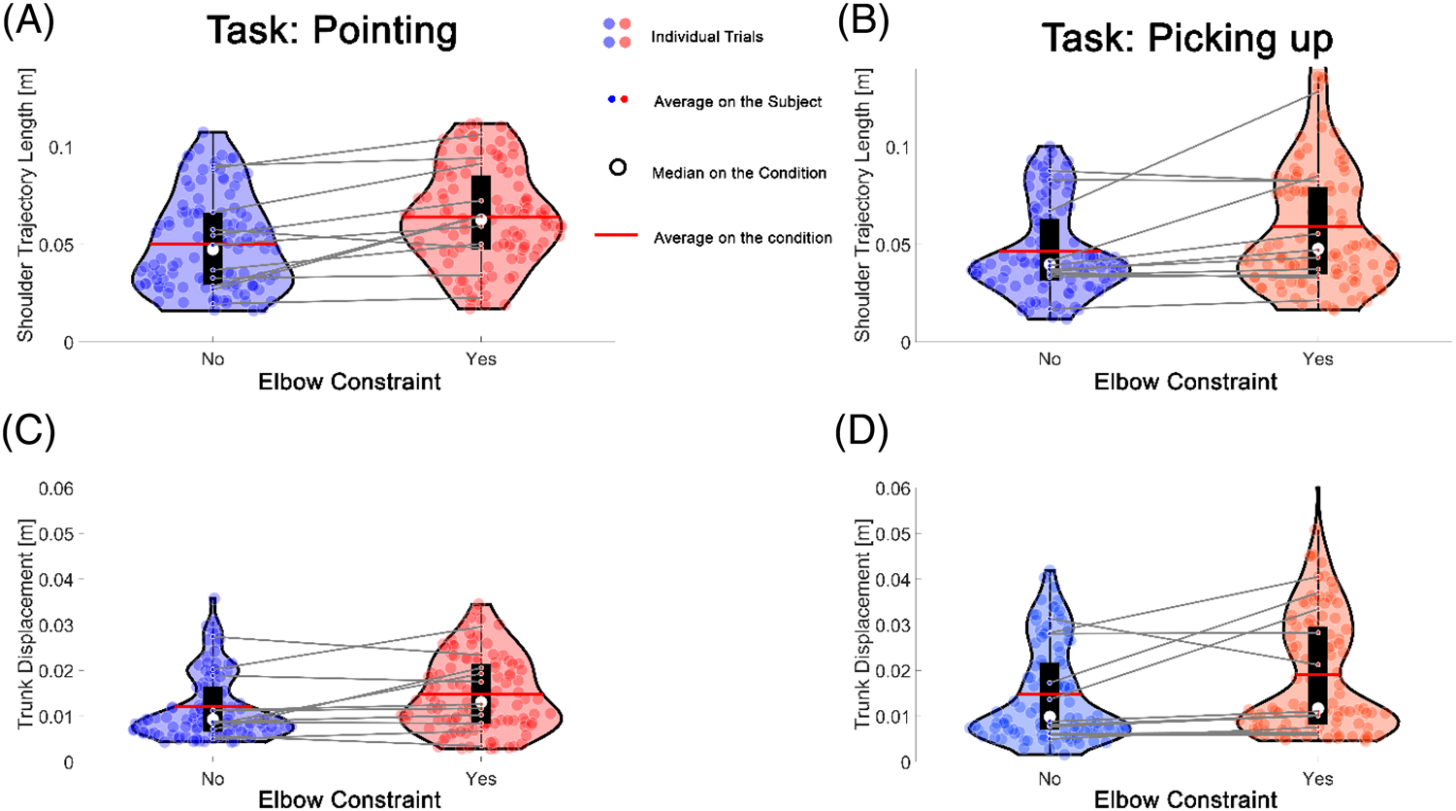
The difference in trunk movements between conditions with and without elbow extension constraint. (**A**,**B**) Comparison of the shoulder trajectory length between elbow constraint conditions for the Pointing and Picking tasks. (**C**,**D**) Comparison of trunk displacement between elbow constraint conditions for the pointing and picking activities.

**Figure 5:**
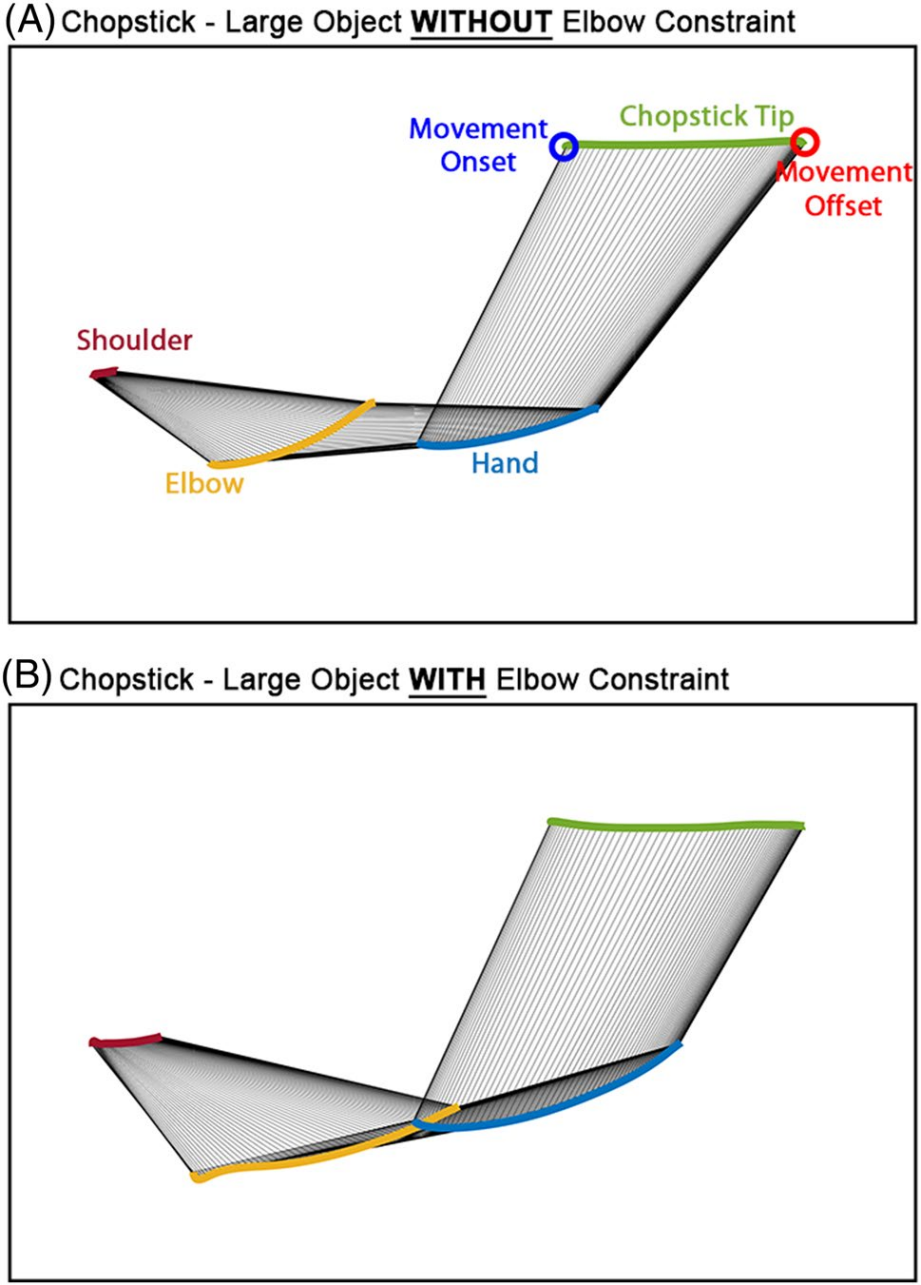
An example demonstrating the shoulder trajectory length difference between conditions with and without elbow constraint. (**A**) Picking up task without elbow extension constraint. (**B**) Picking up task with an elbow extension constraint.

Examination of the summary output for the whole model indicated that the elbow constraint condition, the interaction between task type and difficulty, and the interaction between task difficulty and elbow constraint condition were statistically significant factors of shoulder trajectory length (STL) (Figs. 4 and 5).

STL was, on average, an estimated 0.015 m (m) longer in the elbow constraint condition than in the non-constraint condition ($\widehat{\beta }=0.015, SE=0.003, t=4.76, p<.001$). Neither task type nor difficulty was significant in explaining the STL variances. The interaction term 'task type * task difficulty' revealed a positive coefficient of 0.01, indicating that the combined influence of task type and target size is associated with an increase in shoulder trajectory length.

The complete model for trunk displacement (TD) indicated that the task type, task difficulty, and elbow constraint condition were significant factors. However, TD's differences between different conditions ranged from 2.5 to 3.4 mm (mm), which would not be greater than the noise measurement error.

## Discussion

To our knowledge, this study is the first to compare goal-directed arm reaching kinematics between a pointing task and object pick-up task using a pair of chopsticks. This novel task may serve as a good goal-directed task for future studies where fine-motor hand control is being studied. Our results support that several different task conditions impact goal-directed arm reaching kinematics and compensatory trunk movements in non-disabled young adults. Further, this study is the first to utilize neuromuscular electrical stimulation (NMES) to simulate elbow flexor synergies during goal-directed arm reaches in non-disabled young adults.

### Object pick-up task using a pair of chopsticks

Chopstick operation motor tasks have been used for motor skill learning experiments as they require a high level of hand dexterity^18–23^. Further, chopstick operation tasks have also been used to assess, or improve, fine hand motor skills in individuals after stroke, especially in East Asian countries^24–27^. We employed the object pick-up task using chopsticks to contrast two different task conditions (pointing vs. picking up): one task condition with lower hand dexterity requirement and the other with higher hand dexterity requirement, respectively.

In our experiment, task type was the most crucial factor among the three factors tested that is more impactful for endpoint kinematics of the goal-directed arm reaches. Participants utilized a slower arm reaching movement for the picking up task than the pointing task, which may be related to the accuracy of the hand position at the end of the reaching. This result has been demonstrated in previous studies^7,8^. Carnahan and colleagues tested the effects of unpredictable target position changes on the goal-directed arm reaching kinematics between grasping and pointing movements. They demonstrated that the peak velocity of arm reaching was lower for grasping a target than for pointing at a target. Our finding is aligned with Carnahan and colleagues’ findings. Hand position variability at the end of arm reaches is hypothesized to be associated with a slower arm-reach for picking up objects compared to pointing. In the context of chopstick operation tasks, the object-picking task is more dependent on the positioning of the chopstick tips than the pointing task. Participants may therefore employ slower arm reaches in order to improve the precision of the chopstick tip location and thereby increase the likelihood of completing the task successfully.

Further, slower arm reaching movement would make the online feedback-based adjustment much more manageable. Carnahan and colleagues explained that slower arm reaching movements for grasping tasks than for pointing tasks would also be related to different visual feedback between two tasks^7^. Grafton and colleagues compared different brain activation patterns between pointing and grasping movements in non-disabled adults using positron emission tomography (PET) imaging of regional cerebral blood flow^8^. They found that the left secondary somatosensory cortex (SII) was only recruited while participants performed grasping, not pointing. The different SII activities between the two tasks may indicate that tasks that require more hand dexterity may rely more on visual information to correct errors in goal-directed arm reaching to accurately locate the hand position at the end of the reaching movement. Therefore, sensory association areas, such as SII, would be more activated in tasks requiring higher hand dexterity than a simple pointing task to integrate the somatosensory and visual information. We hypothesize that participants in our study utilized visual input as a means to control the goal-directed arm reaches. Visual feedback plays a critical role in enhancing the precision of the chopstick tip position at the end of arm reaches; furthermore, it can inform the motor control methods employed during arm reaches. Neuroimaging research is required in the future to better comprehend how brain activity varies across motor tasks and how this variation affects the kinematics of arm reaching.

In our study, slower arm reaching movements in the picking up task than in the pointing task may indicate that participants utilized more feedback-based control in the picking up task than in the pointing task. Other goal-directed arm reaching kinematic data in our study support that participants relied more on feedback-based control in the picking up task than the pointing task. The amplitude of peak tangential velocity (PV) is primarily determined through pulse-height and pulse-width control mechanisms of acceleration^9^. Participants showed a shorter TTPA (pulse-width control) and a less peak acceleration (PA—pulse-height control) of goal-directed arm reaches in the pointing task than in the picking up task. These results may indicate that participants relied on more feedforward control of goal-directed arm reaches in the pointing task and more feedback-based control in the picking up task. Participants also utilized a more prolonged deceleration phase of goal-directed arm reaching movement when they performed the picking-up task rather than the pointing task. The mixed effects model for the relative time to peak velocity (TTPV%) revealed that the proportion of acceleration phase of the reaching was significantly less in the picking up task than in the pointing task. This finding is consistent with a prior investigation conducted by Carnahan and colleagues^7^ which demonstrated that the duration required to reach the peak velocity was comparable for both pointing and grasping activities. However, the deceleration time for the reaching movement was longer in the grasping task.

As the picking up task using a pair of chopsticks necessitates greater hand position precision at the end of the reaching movement, participants may subsequently place greater reliance on online feedback-based control of the arm reaching movement. Once more, the implementation of feedback-based control would enhance the precision of the end-effector (chopstick tips) position at the end of the reaching movement, hence facilitating the effective manipulation of an object. In order to increase end-effector position accuracy (i.e., reduce spatial error) at the completion of arm reaches, non-disabled young adults may utilize increased online feedback-based control of goal-directed arm reaching movement for activities requiring greater hand dexterity in light of these findings.

Further, movement smoothness difference between two tasks would also indicate more online feedback-based control of arm reaching in the picking up task than in the pointing task. Goal-directed arm reaching movements were jerkier in the picking up task than in the pointing task. A jerkier goal-directed arm reaching movement may indicate utilization of more corrective sub-movements^28^, indicating feedback-based adjustment of the hand position during reaching movement.

Altogether, our results support that participants utilized different goal-directed arm reaching control mechanisms when they performed different tasks requiring different levels of hand dexterity. When participants performed a manipulative task that required higher hand dexterity, they utilized more online feedback-based control of reaching movements. In comparison, they utilized more feedforward control in a simple pointing task that required less hand dexterity. Our preliminary data with two chronic stroke survivors with mild upper extremity motor impairment showed similar results. Therefore, future studies are required to test how different tasks with different hand dexterity requirements would impact motor control strategies in individuals after stroke.

### Trunk compensatory movements during goal-directed arm reaches

Trunk movements during goal-directed arm reaching are subtle in non-disabled young adults when they reach an object within their maximal arm reaching extent. Therefore, we did not expect significant trunk movements during the task performance in non-disabled young participants when there was no constraint to elbow extension. We utilized neuromuscular electrical stimulation (NMES) on the elbow flexors of the performing arm to constrain elbow extension. The reason why we used the NMES for elbow extension constrain is that this would best simulate the elbow extension constrain in individuals after stroke. Many stroke survivors, specifically those with moderate to severe upper extremity motor impairments, experience upper extremity flexor synergies with increased muscle tone (hypertonicity) or spasticity of elbow flexors. In this case, the flexor hypertonicity or spasticity increases the resistance to the elbow extension. NMES on the antagonist functions as resistance to the intended movement induced by the agonist contraction, as demonstrated in a prior work^29^.

Further, the reciprocal inhibition from the elbow flexor to the elbow extensor also impact the elbow extension active range of motion^30,31^. While there have been several studies investigating the effects of a mechanical constrain of joint movement on movement strategies^32,33^, the mechanical joint constraint is not appropriate to test our hypothesis as participants may utilize different trunk compensation anticipating the restriction from the visual information of the constraint. On the other hand, our approach does not influence the participant’s action at rest, and also generate the reciprocal inhibition of the elbow extensor from the NMES-induced elbow flexor activities.

This elbow extension constraint condition was used to determine how non-disabled young adults compensate for the constraint to perform the task. The elbow constraint condition was a significant factor in determining the endpoint kinematics of goal-directed arm reaching movement. As expected, this condition was also a significant factor in shoulder trajectory length (STL) and trunk displacement (TD), while task type and difficulty were not independently significant factors.

STL represents the travel distance of the shoulder landmark of the performing arm. This measure quantifies trunk movements in all three directions (Anterior–posterior [A–P], medial–lateral [M-L], and rotation), while the TD represent the trunk movement only in the A–P direction.

Participants showed a significantly more extended STL in elbow-restricted conditions than in non-restricted conditions. Although this difference in the STL between the two conditions was statistically significant, the difference in STL was only 1.5 cm between the two conditions. This difference would not be functionally meaningful. We compared these results with the STL during the same task performance from two chronic stroke survivors with mild upper extremity motor impairment (See Supplemental Table S1 for demographics of two stroke survivors). In fact, the STL was 6.25 cm on average with the elbow constraint in non-disabled young adults, while the STL was 11.117 cm on average in two participants with post-stroke mild upper extremity motor impairment.

Although the 1.5 cm difference in the STL between the two conditions is relatively small, this difference is statistically more significant than the difference in the TD, which was only 2 mm (mm) on average. This finding may indicate that the trunk compensation during the goal-directed arm reaches with elbow extension constraint would mainly be the trunk rotation, not the anterior displacement (i.e., trunk flexion).

Our findings may indicate that the limited active elbow extension partially explains trunk compensation during goal-directed arm reaching in chronic stroke survivors. In the preliminary data analysis, two chronic stroke survivors had mild upper extremity motor impairment, and their active range of motion of the paretic elbow extension was normal. It is possible that chronic stroke survivors would utilize trunk compensation during goal-directed arm reaches even when they accomplished full restitution of the voluntary elbow extension, as trunk compensation would be a learned motor behavior^34^.

**Figure 6:**
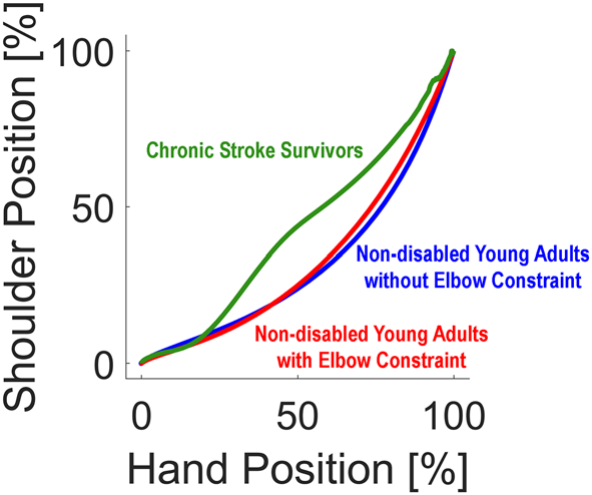
Normalized hand-shoulder position plot.

We performed an additional post-hoc analysis to determine the timing difference in the trunk movement onset. We compared the hand-shoulder position plots^34^ for each condition with normalization for inter-individual comparison. The normalized hand-shoulder position plot showed a different onset timing of the trunk movements (i.e., slopes of the line) between non-disabled young adults and chronic stroke survivors. Trunk movement, represented by the shoulder landmark, was initiated earlier in chronic stroke survivors than in non-disabled young adults (Fig. 6). The trajectory of stroke survivors reveals a noteworthy distinction in the association between hand position and shoulder movement when compared to non-disabled young adults. Particularly, the movement of the shoulder marker, indicative of compensatory trunk movements, manifests in the early phase of arm reaching (prior to 50% of the arm reaching movement) for stroke survivors. In contrast, non-disabled young adults, under elbow constraint, exhibit more shoulder marker movement compared to no elbow constraint condition in the later stages of arm reaches. This disparity suggests that stroke survivors employ a feedforward control strategy for trunk movement, suggesting an anticipatory adjustment, while non-disabled adults demonstrate trunk movement primarily driven by feedback-based control. This observation underscores the adaptive strategies developed by stroke survivors in motor planning and execution, highlighting the potential influence of learned behavior on the temporal aspects of trunk involvement in reaching tasks. On the other hand, non-disabled young adults in the study engaged compensatory trunk movement when the elbow extension was restricted as a result of feedback-based online control.

### Limitations

This study only includes 11 non-disabled young adults. To overcome issues posed by the small sample size, we used data from each individual trial, not the average data for each participant, in the linear mixed-effects modeling. The inter-individual variability and inter-trial variability were considered in the model by adding random effects terms of the participant and trial. Due to limited number of chronic stroke participants, we were unable to perform comparison of kinematic variables between two participant populations. Future study with a large sample size is required to confirm our findings in chronic stroke survivors. Another limitation was that participants had various chopstick usage experiences and skill levels. We compared mixed-effect models with and without chopstick usage experiences and self-efficacy as random variables. The result showed that adding these random variables did not significantly improve the model. Therefore, we assume that the previous chopstick usage experience and chopstick skill self-efficacy did not impact participants' motor task performance. Future studies with a larger sample size are needed to corroborate our findings.

We utilized shoulder trajectory length to compare trunk movements during goal-directed arm reaches. This trunk movement measure has not been tested for its validation and reliability. However, our preliminary results showed that the STL is more sensitive than trunk displacement in chronic stroke survivors to quantify the trunk movements during goal-directed arm reaches. Future studies are needed to test its validity and reliability.

Another limitation of our study is that we did not incorporate the Index of Difficulty (ID) from Fitt’s law^35^ or the modified ID from recent studies, such as those by Lucchese and colleagues^36^, to quantify task difficulty explicitly. While these metrics are valuable for continuous and repetitive pointing or grasping motor tasks, our discrete motor tasks, characterized by distinct arm reaching movement onsets and offsets, posed challenges in the direct application of the ID equations. As a result, we relied on the target size as an alternative measure of task difficulty in our statistical analyses. While this choice allowed us to explore the impact of target size on motor performance, it is essential to acknowledge that our approach diverged from the traditional application of the ID metrics in motor control studies. Future research could potentially explore adaptations of these metrics to discrete motor tasks to provide a more comprehensive evaluation of task difficulty in similar contexts.

## Conclusion

This study illuminates the different goal-directed arm reaching control strategies employed by non-disabled young adults when performed motor tasks of varying hand dexterity requirements. Notably, our investigation affirms that imposing an elbow extension constraint can influence the motor control strategies governing goal-directed arm reaches in this population. These results underscore the imperative consideration of specific motor task conditions, particularly the intricacies of hand dexterity demand, in the assessment and intervention of arm and hand motor function.

### Supplementary Information

Supplementary Information.

## Data Availability

The datasets used and/or analyzed during the current study available from the corresponding author on reasonable request.
