# Supplementary material for: Impact of motor task conditions on end-point kinematics and trunk movements during goal-directed arm reach
Source: Sci Rep. 2024 Feb 24;14:4520. doi: 10.1038/s41598-024-54723-4 (PMC10894230; doi:10.1038/s41598-024-54723-4)

**Supplemental Table 1. Demographics of Stroke Participants.**

| Demographic Variables | | Stroke Survivor 1 | Stroke Survivor 2 |
| --- | --- | --- | --- |
| Age (years) | | 63 | 73 |
| Sex | | Female | Male |
| Race | | White | White |
| Hand Dominance | | Right | Right |
| Affected Side | | Right | Right |
| Previous Chopstick Usage | | No | Yes |
| Chopstick usage frequency within 6 months | | 0 | Less than 5 times per year |
| Self-Efficacy | | | |
|  | Affected hand - Large object | 0 | 78 |
|  | Non-affected hand - Large object | 52 | 1 |
|  | Affected hand - Small object | 0 | 4 |
|  | Non-affected hand - Small object | 51 | 3 |
| Upper Extremity Fugl-Meyer Assessment | | 55 | 59 |

**Supplemental Table 2. Linear Mixed Effects model comparisons for Movement Duration.**

| Model # | Equation |
| --- | --- |
| lme1_MD | MD~Task_type + Target_size + Stim_type + Task_type*Target_size + Task_type*Stim_type + Target_size*Stim_type |
| lme2_MD | MD~Task_type + Target_size + Stim_type + Task_type*Target_size + Task_type*Stim_type + Target_size*Stim_type + (1\|ID) |
| lme3_MD | MD~Task_type + Target_size + Stim_type + Task_type*Target_size + Task_type*Stim_type + Target_size*Stim_type + (1\|ID) + (1\|Trial) |
| lme4_MD | MD~Task_type + Target_size + Stim_type + Task_type*Target_size + Task_type*Stim_type + Target_size*Stim_type + (1\|ID) + (1+Task_type\|ID) + (1\|Trial) + (1+Task_type\|Trial) |
| lme5_MD | MD~Task_type + Target_size + Stim_type + Task_type*Target_size + Task_type*Stim_type + Target_size*Stim_type + (1\|ID) + (1+Target_size\|ID) + (1\|Trial) + (1+Target_size\|Trial) |
| lme6_MD | MD~Task_type + Target_size + Stim_type + Task_type*Target_size + Task_type*Stim_type + Target_size*Stim_type + (1\|ID) + (1+Stim_type\|ID) + (1\|Trial) + (1+Stim_type\|Trial) |
| lme7_MD | MD ~ 1 + Task_type*Target_size + Task_type*Stim_type + Target_size*Stim_type + (1 \| ID) + (1 + Task_type \| ID) + (1 + Target_size \| ID) + (1 + Stim_type \| ID) + (1 \| Trial) + (1 + Task_type \| Trial) + (1 + Target_size \| Trial) + (1 + Stim_type \| Trial) |
| lme8_MD | MD ~ 1 + Task_type*Target_size + Task_type*Stim_type + Target_size*Stim_type + (1 \| ID) + (1 + Task_type \| ID) + (1 + Target_size \| ID) + (1 + Stim_type \| ID) + (1 \| Trial) + (1 + Task_type \| Trial) + (1 + Target_size \| Trial) + (1 + Stim_type \| Trial) + (1 \| Chopstick_use) |

**Supplemental Table 3. Linear mixed effects model comparison for Movement Duration using ANOVA.**

| Models | DF | AIC | BIC | LogLik | LRStat | deltaDF | pValue |
| --- | --- | --- | --- | --- | --- | --- | --- |
| lme1_MD | 8 | -558.321 | -520.229 | 287.1606 |  |  |  |
| lme2_MD | 9 | -916.802 | -873.948 | 467.401 | 360.4809 | 1 | **0***** |
|  | | | | | | | |
| Models | DF | AIC | BIC | LogLik | LRStat | deltaDF | pValue |
| lme2_MD | 9 | -916.802 | -873.948 | 467.401 |  |  |  |
| lme3_MD | 10 | -936.402 | -888.787 | 478.2012 | 21.60027 | 1 | **3.36E-06***** |
|  | | | | | | | |
| Models | DF | AIC | BIC | LogLik | LRStat | deltaDF | pValue |
| lme3_MD | 10 | -936.402 | -888.787 | 478.2012 |  |  |  |
| lme4_MD | 16 | -1024.4 | -948.219 | 528.202 | 100.0016 | 6 | **0***** |
|  | | | | | | | |
| Models | DF | AIC | BIC | LogLik | LRStat | deltaDF | pValue |
| lme3_MD | 10 | -936.402 | -888.787 | 478.2012 |  |  |  |
| lme5_MD | 16 | -939.722 | -863.536 | 485.8608 | 15.3192 | 6 | **0.017914*** |
|  | | | | | | | |
| Models | DF | AIC | BIC | LogLik | LRStat | deltaDF | pValue |
| lme3_MD | 10 | -936.402 | -888.787 | 478.2012 |  |  |  |
| lme6_MD | 16 | -973.936 | -897.751 | 502.9679 | 49.53337 | 6 | **5.83E-09***** |
|  | | | | | | | |
| Models | DF | AIC | BIC | LogLik | LRStat | deltaDF | pValue |
| lme3_MD | 10 | -936.402 | -888.787 | 478.2012 |  |  |  |
| lme7_MD | 28 | -1083.5 | -950.178 | 569.7512 | 183.1001 | 18 | **0***** |
|  | | | | | | | |
| Model | DF | AIC | BIC | LogLik | LRStat | deltaDF | pValue |
| lme7_MD | 28 | -1083.5 | -950.178 | 569.7512 |  |  |  |
| lme8_MD | 29 | -1081.5 | -943.417 | 569.7512 | 1.82E-12 | 1 | 1 |

**p < .05, **p < .01, ***p < .001*

**Supplemental Table 4. ANOVA statistics of Linear mixed effects models**

| Movement Duration (MD) | | | | |
| --- | --- | --- | --- | --- |
| Term | FStat | DF1 | DF2 | pValue |
| (Intercept) | 315.82 | 1 | 857 | 0.000 |
| Task_type | 63.54 | 1 | 857 | 0.000 |
| Target_size | 0.95 | 1 | 857 | 0.331 |
| Stim_type | 5.91 | 1 | 857 | 0.015 |
| Task_type:Target_size | 51.31 | 1 | 857 | 0.000 |
| Task_type:Stim_type | 12.26 | 1 | 857 | 0.000 |
| Target_size:Stim_type | 1.62 | 1 | 857 | 0.203 |
|  |  |  |  |  |
| Peak Velocity (PV) | | | | |
| Term | FStat | DF1 | DF2 | pValue |
| (Intercept) | 646.62 | 1 | 857 | 0.000 |
| Task_type | 34.61 | 1 | 857 | 0.000 |
| Target_size | 0.36 | 1 | 857 | 0.550 |
| Stim_type | 12.98 | 1 | 857 | 0.000 |
| Task_type:Target_size | 48.33 | 1 | 857 | 0.000 |
| Task_type:Stim_type | 16.82 | 1 | 857 | 0.000 |
| Target_size:Stim_type | 0.05 | 1 | 857 | 0.816 |
|  |  |  |  |  |
| Absolute Time to peak velocity (aTTPV) | | | | |
| Term | FStat | DF1 | DF2 | pValue |
| (Intercept) | 873.71 | 1 | 857 | 0.000 |
| Task_type | 9.44 | 1 | 857 | 0.002 |
| Target_size | 1.54 | 1 | 857 | 0.215 |
| Stim_type | 12.46 | 1 | 857 | 0.000 |
| Task_type:Target_size | 15.22 | 1 | 857 | 0.000 |
| Task_type:Stim_type | 8.71 | 1 | 857 | 0.003 |
| Target_size:Stim_type | 1.43 | 1 | 857 | 0.232 |
|  |  |  |  |  |
| Relative Time to peak velocity (rTTPV) | | | | |
| Term | FStat | DF1 | DF2 | pValue |
| (Intercept) | 634.11 | 1 | 857 | 0.000 |
| Task_type | 61.43 | 1 | 857 | 0.000 |
| Target_size | 0.37 | 1 | 857 | 0.545 |
| Stim_type | 3.56 | 1 | 857 | 0.060 |
| Task_type:Target_size | 12.88 | 1 | 857 | 0.000 |
| Task_type:Stim_type | 0.31 | 1 | 857 | 0.576 |
| Target_size:Stim_type | 0.27 | 1 | 857 | 0.602 |

| Dimensionless Log Jerkiness(DMLJ) | | | | |
| --- | --- | --- | --- | --- |
| Term | FStat | DF1 | DF2 | pValue |
| (Intercept) | 4084.67 | 1 | 857 | 0.000 |
| Task_type | 62.91 | 1 | 857 | 0.000 |
| Target_size | 0.33 | 1 | 857 | 0.566 |
| Stim_type | 2.34 | 1 | 857 | 0.127 |
| Task_type:Target_size | 40.40 | 1 | 857 | 0.000 |
| Task_type:Stim_type | 8.17 | 1 | 857 | 0.004 |
| Target_size:Stim_type | 0.94 | 1 | 857 | 0.332 |
|  |  |  |  |  |
| Shoulder Trajectory Length (STL) | | | | |
| Term | FStat | DF1 | DF2 | pValue |
| (Intercept) | 56.07 | 1 | 857 | 0.000 |
| Task_type | 0.95 | 1 | 857 | 0.331 |
| Target_size | 2.99 | 1 | 857 | 0.084 |
| Stim_type | 26.20 | 1 | 857 | 0.000 |
| Task_type:Target_size | 31.77 | 1 | 857 | 0.000 |
| Task_type:Stim_type | 3.61 | 1 | 857 | 0.058 |
| Target_size:Stim_type | 28.49 | 1 | 857 | 0.000 |
|  |  |  |  |  |
| Trunk Displacement (TD) | | | | |
| Term | FStat | DF1 | DF2 | pValue |
| (Intercept) | 32.70 | 1 | 857 | 0.000 |
| Task_type | 3.48 | 1 | 857 | 0.062 |
| Target_size | 3.62 | 1 | 857 | 0.057 |
| Stim_type | 2.81 | 1 | 857 | 0.094 |
| Task_type:Target_size | 2.66 | 1 | 857 | 0.103 |
| Task_type:Stim_type | 0.38 | 1 | 857 | 0.536 |
| Target_size:Stim_type | 0.25 | 1 | 857 | 0.615 |

**Supplemental Table 5. Coefficients estimates for linear mixed effects models**

| MD | | | | | |
| --- | --- | --- | --- | --- | --- |
| Name | Estimate | SE | tStat | DF | pValue |
| (Intercept) | 0.57 | 0.03 | 17.77 | 857 | 0.000 |
| Task_type_2 | 0.27 | 0.03 | 7.97 | 857 | 0.000 |
| Target_size_2 | 0.02 | 0.02 | 0.97 | 857 | 0.331 |
| Stim_type_2 | 0.06 | 0.03 | 2.43 | 857 | 0.015 |
| Task_type_2:Target_size_2 | -0.11 | 0.02 | -7.16 | 857 | 0.000 |
| Task_type_2:Stim_type_2 | -0.06 | 0.02 | -3.50 | 857 | 0.000 |
| Target_size_2:Stim_type_2 | -0.02 | 0.02 | -1.27 | 857 | 0.203 |
|  |  |  |  |  |  |
| PV | | | | | |
| Name | Estimate | SE | tStat | DF | pValue |
| (Intercept) | 0.78 | 0.03 | 25.43 | 857 | 0.000 |
| Task_type_2 | -0.15 | 0.03 | -5.88 | 857 | 0.000 |
| Target_size_2 | -0.01 | 0.01 | -0.60 | 857 | 0.550 |
| Stim_type_2 | -0.06 | 0.02 | -3.60 | 857 | 0.000 |
| Task_type_2:Target_size_2 | 0.06 | 0.01 | 6.95 | 857 | 0.000 |
| Task_type_2:Stim_type_2 | 0.03 | 0.01 | 4.10 | 857 | 0.000 |
| Target_size_2:Stim_type_2 | 0.00 | 0.01 | 0.23 | 857 | 0.816 |
|  |  |  |  |  |  |
| TTPV | | | | | |
| Name | Estimate | SE | tStat | DF | pValue |
| (Intercept) | 0.23 | 0.01 | 29.56 | 857 | 0.000 |
| Task_type_2 | 0.03 | 0.01 | 3.07 | 857 | 0.002 |
| Target_size_2 | 0.01 | 0.01 | 1.24 | 857 | 0.215 |
| Stim_type_2 | 0.04 | 0.01 | 3.53 | 857 | 0.000 |
| Task_type_2:Target_size_2 | -0.02 | 0.01 | -3.90 | 857 | 0.000 |
| Task_type_2:Stim_type_2 | -0.02 | 0.01 | -2.95 | 857 | 0.003 |
| Target_size_2:Stim_type_2 | -0.01 | 0.01 | -1.20 | 857 | 0.232 |
| TTPV2 | | | | | |
| Name | Estimate | SE | tStat | DF | pValue |
| (Intercept) | 42.18 | 1.68 | 25.18 | 857 | 0.000 |
| Task_type_2 | -9.48 | 1.21 | -7.84 | 857 | 0.000 |
| Target_size_2 | 0.66 | 1.09 | 0.61 | 857 | 0.545 |
| Stim_type_2 | 1.93 | 1.02 | 1.89 | 857 | 0.060 |
| Task_type_2:Target_size_2 | 2.90 | 0.81 | 3.59 | 857 | 0.000 |
| Task_type_2:Stim_type_2 | 0.45 | 0.81 | 0.56 | 857 | 0.576 |
| Target_size_2:Stim_type_2 | -0.42 | 0.81 | -0.52 | 857 | 0.602 |
|  |  |  |  |  |  |
| DMLJ | | | | | |
| Name | Estimate | SE | tStat | DF | pValue |
| (Intercept) | -13.28 | 0.21 | -63.91 | 857 | 0.000 |
| Task_type_2 | -1.55 | 0.20 | -7.93 | 857 | 0.000 |
| Target_size_2 | -0.07 | 0.12 | -0.57 | 857 | 0.566 |
| Stim_type_2 | -0.18 | 0.12 | -1.53 | 857 | 0.127 |
| Task_type_2:Target_size_2 | 0.62 | 0.10 | 6.36 | 857 | 0.000 |
| Task_type_2:Stim_type_2 | 0.28 | 0.10 | 2.86 | 857 | 0.004 |
| Target_size_2:Stim_type_2 | 0.09 | 0.10 | 0.97 | 857 | 0.332 |
|  |  |  |  |  |  |
| STL | | | | | |
| Name | Estimate | SE | tStat | DF | pValue |
| (Intercept) | 0.05 | 0.01 | 7.49 | 857 | 0.000 |
| Task_type_2 | 0.00 | 0.00 | -0.97 | 857 | 0.331 |
| Target_size_2 | 0.01 | 0.00 | 1.73 | 857 | 0.084 |
| Stim_type_2 | 0.01 | 0.00 | 5.12 | 857 | 0.000 |
| Task_type_2:Target_size_2 | 0.01 | 0.00 | 5.64 | 857 | 0.000 |
| Task_type_2:Stim_type_2 | 0.00 | 0.00 | -1.90 | 857 | 0.058 |
| Target_size_2:Stim_type_2 | -0.01 | 0.00 | -5.34 | 857 | 0.000 |

| TD | | | | | |
| --- | --- | --- | --- | --- | --- |
| Name | Estimate | SE | tStat | DF | pValue |
| (Intercept) | 0.01 | 0.00 | 5.72 | 857 | 0.000 |
| Task_type_2 | 0.00 | 0.00 | 1.87 | 857 | 0.062 |
| Target_size_2 | 0.00 | 0.00 | 1.90 | 857 | 0.057 |
| Stim_type_2 | 0.00 | 0.00 | 1.68 | 857 | 0.094 |
| Task_type_2:Target_size_2 | 0.00 | 0.00 | -1.63 | 857 | 0.103 |
| Task_type_2:Stim_type_2 | 0.00 | 0.00 | 0.62 | 857 | 0.536 |
| Target_size_2:Stim_type_2 | 0.00 | 0.00 | -0.50 | 857 | 0.615 |

**Supplemental Figure 1. Final linear mixed effects model fit.**


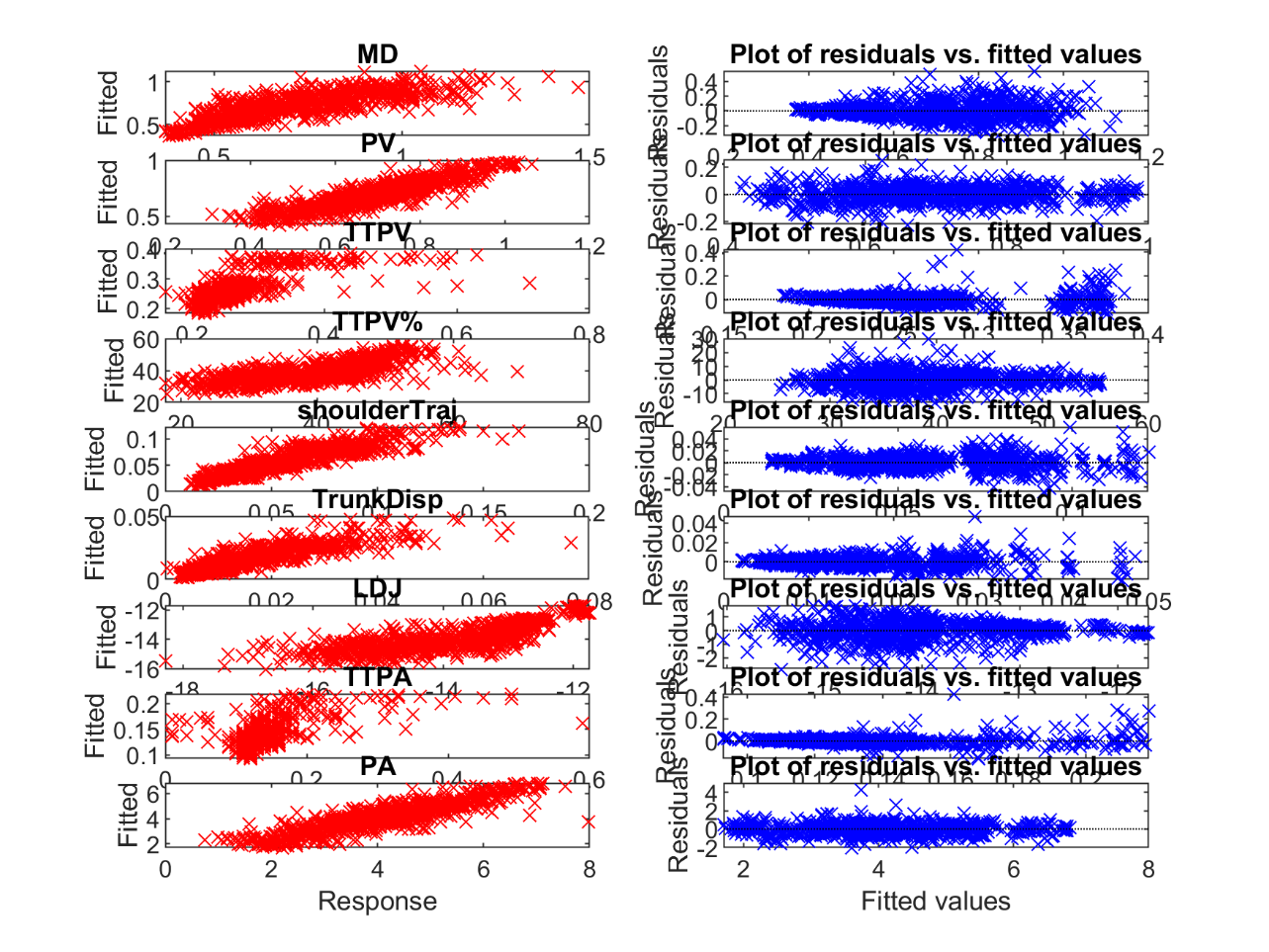

Supplement: Supplementary file 1 — Supplementary Information. [file 41598_2024_54723_MOESM1_ESM.docx]
